# Plasma Cell Granuloma of the Thyroid: A Conservative Approach to a Rare Condition and Review of the Literature

**DOI:** 10.4061/2010/840469

**Published:** 2010-02-07

**Authors:** W. A. Barber, M. Fernando, D. R. Chadwick

**Affiliations:** ^1^Department of General Surgery, Chesterfield Royal Hospital, Calow, Chesterfield S44 5BL, UK; ^2^Department of Histopathology, Sheffield Teaching Hospitals NHS Foundation Trust, Royal Hallamshire Hospital, Glossop Road, Sheffield S10 2JF, UK

## Abstract

*Introduction*. We present a case of an 89-year-old female who attended our surgical endocrine clinic with a 3-month history of a left-sided neck lump. There was no past medical history of thyroid disease. *Methods*. Following examination and further investigation, including core biopsy, a diagnosis of plasma cell granuloma of the thyroid was made. Biochemical testing of thyroid function and Thyroid Peroxidase Antibody was in-keeping with an associated Hashimoto's thyroiditis. *Results*. The patient was treated conservatively with thyroxine and regularly seen in clinic. TSH levels improved and the lump showed signs of regression. *Conclusion*. Plasma cell granuloma of the thyroid is rare with only 16 previously reported cases. We present a new approach to management without the use of surgery or steroids. The literature is reviewed comparing clinico-pathological features and management of other reported cases.

## 1. Case Report

An 89-year-old female presented with a 3-month history of a left-sided neck lump. The lump had been steadily increasing in size during this time. There was no history of shortness of breath, dysphagia, or stridor and no history of voice change. The patient had a past medical history of vascular dementia, hypertension, and B12 deficiency secondary to pernicious anaemia. Regular medications included aspirin, bendrofluazide, and 3 monthly injections of hydroxocobalamin. There was no past medical history of thyroid disease or neck irradiation and no family history of autoimmune disease.

On examination the patient was frail and clinically euthyroid. Examination of the neck revealed a large firm, irregular mass in the upper pole of the left thyroid lobe with a background of multinodular goitre. The lump measured 6.1 cm × 5.5 cm with calipers on presentation. There was no evidence of lymphadenopathy and the trachea was central with no signs of stridor.

Initial assessment was suggestive of lymphoma or poorly differentiated carcinoma. In order to increase diagnostic accuracy, a needle core biopsy was taken rather than fine needle aspiration. Two passes were made using a 14-guage needle. TFTs were checked revealing a TSH of 17.6 *μ*IU/L (0.4–5.5 *μ*IU/L) with a Thyroid Peroxidase Antibody (TPA) of 557 IU/ml (0–50 IU/ml) and free T4 of 12.5 pmol/L (11–26 pmol/L). Full blood count, liver function tests, and urea and electrolytes were all in the normal range. These results were in-keeping with hypothyroidism due to Hashimoto's thyroiditis.

**Figure 1:**
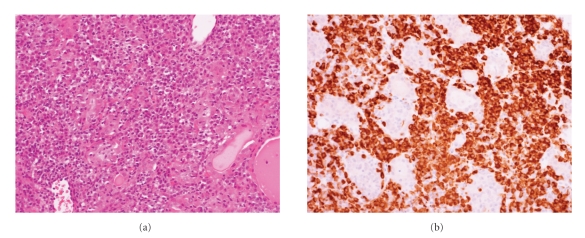
Core biopsy showing plasma cells confirmed with staining for CD79a ((a) H&E (b) CD79a both ×200).

**Figure 2:**
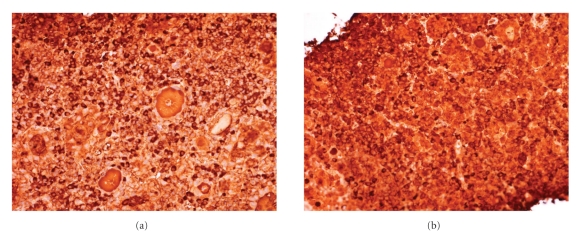
Staining for kappa (a) and lambda (b) light chains to confirm polyclonality (both ×200).

Two core biopsies both measuring 15 mm were obtained for histological examination. This showed a heavy plasma cell infiltrate and admixed B- and T-lymphocytes (Figure 1). The plasma cell infiltrate was polyclonal (Figure 2) and expressed CD79a, CD138, and MUM-1. There was no evidence of anaplastic carcinoma or other primary thyroid carcinoma. There were no morphological features to suggest Riedels thyroiditis.

The histological findings were therefore consistent with a plasma cell granuloma of the thyroid with underlying Hashimoto's thyroiditis.

Due to patient frailty and comorbidities, operative intervention was deemed inappropriate. The patient was regularly reviewed in the clinic having been started on Thyroxine. TSH levels improved with modification of T4 dosage. The neck lump remained static for several months until eventually showing signs of regression. The lump measured 4.5 cm × 3 cm 10 months after presentation and 8 months following start of treatment with thyroxine. The patient remained asymptomatic with respect to breathing and swallowing.

## 2. Discussion

Plasma cell granuloma of the thyroid gland is rare with only 16 previously reported cases. It predominantly affects women with the majority of cases reporting patients over the age of 35 years. Plasmacytoma of the thyroid is more common and the two can be differentiated histologically by assessing for clonality.

Plasma cell granuloma (PCG) is well documented in the literature, first being described in 1973 by Bahadori and Liebow [1]. It is a nonmalignant lesion characterised by proliferation of polyclonal plasma cells with varying degrees of myofibroblastic proliferation [2]. The polyclonal nature is important in distinguishing PCG from plasmacytoma. Immunohistochemistry can also be used to demonstrate the polyclonality of the plasma cells. Lesions of this type are mainly found in the lungs with other recorded cases occurring in the liver [3], stomach [4], pancreas [5], bladder [6], and kidney [7]. Hurthle cell metaplasia found on histology has been documented in some cases [8–10] of PCG of the thyroid but this is not universal. Macroscopically, there are also some variations in the literature but the lesions are usually firm with a white/grey colour. Often the specimen has been as part of a lobectomy or total thyroidectomy. 

The aetiology of plasma cell granulomas is not completely understood. It has been suggested that it may be secondary to a chronic inflammatory process causing abnormalities of plasma cell differentiation. Many of the cases of thyroid plasma cell granulomas demonstrate an association with an autoimmune disease such as Hashimoto's thyroiditits and diabetes mellitus [10–14]. This is largely anecdotal and, although there is no strong evidence to link the two disease processes, it can be supported by evidence of the cellular infiltrate expressing antithyroid peroxidase antibodies and response to immunosuppressant medications.

Treatment of these lesions varies in the literature with the majority of patients undergoing some form of surgical intervention with either total/subtotal thyroidectomy or lobectomy (Table 1). Corticosteroid usage and other immunosuppressive therapies such as cyclophosphamide and azathioprine have also been used to treat these lesions with some degree of improvement. In our case, we have shown that these benign lesions can resolve spontaneously without the need for unnecessary surgery or medications with potentially significant side effects. We do, however, appreciate that surgical intervention may be a necessity in a case of a large, rapidly increasing nodule that is compromising a patients airway or is associated with significant symptoms. However, if PCG is confirmed histologically, for example, on core biopsy as in our patient, with no debilitating symptoms, it is reasonable to observe these cases without any intervention either surgical or medical other than treatment of any underlying thyroid dysfunction.

## Figures and Tables

**Table 1:** Clinical and pathological features of reported cases of plasma cell granuloma of the thyroid.

Paper	Age/Sex	Presentation	Thyroid function	Autoimmunity	Pathology	Treatment
Chan et al. 1986 [15]	35 F	Neck lump, right lobe nodule. Mild tracheal compression	Euthyroid	No	3 cm white, round nodule. Plasma cell aggregates. Hurtle cells absent. Polyclonal pattern on staining	Right hemithyroidectomy
De Mascarel et al. 1989 [16]	35 F	3 cm nodule in left lobe	Euthyroid	No	2.2 cm firm lesion. Fibrous tissue with polyclonal plasma cells	Thyroidectomy
Ferrer-Garcia et al. 2004 [11]	41 M	Goiter	Hypothyroid	Hashimoto's	Polyclonal plasma cells with evidence of Hashimoto's thyroiditis.	FNA inconclusive. Total thyroidectomy
Fontenot et al. 2008 [17]	55 F	Enlarging neck swelling, with compressive symptoms	Hypothyroid	No	Firm, fibrotic lesion. Polyclonal plasma cells with the expression of both kappa and lambda light chains	Thyroidectomy
Holck, 1981 [18]	70 F	Neck swelling with breathing difficulties. Right lobe, 3 cm nodule on examination	Hypothyroid	No	Obliteration of parenchyma with mature plasma cells. No Hurtle cell changes	Subtotal thyroidectomy
Kojima et al. 2009 [19]	75 F	Painless left-sided neck swelling	Euthyroid	No	Inflammatory pseudotumour (IPT). Predominantly fibrohistiocytic. Vimentin and CD68 +ve.	Lobectomy
Kriegl et al. 2007 [12]	50 M	Thyroid enlargement with dysphagia	Euthyroid	Hashimoto's	Polyclonal plasma cells with associated Hashimoto's thyroiditis. EBV and HHV8 DNA negative.	Subtotal thyroidectomy
Laurent et al. 2004 [13]	35 F	Dysphagia and asthenia. Normal thyroid on examination. Later painful enlargement of thyroid with signs of tracheal compression	Hypothyroid	?Hashimoto's (↑ antimicrosomal antibodies, anti-TPO positive)	Numerous plasma cells, macrophages and T lymphocytes and B lymphocytes. Plasma cells polyclonal	Methylprednisolone initially without response. Unable to excise due to fibrosis, biopsy taken. IV methylprednisolone given followed by IV cyclophosphamide and oral azathioprine for 6 months
Li Voon Chong et al. 2001 [20]	29 M	Neck tenderness, dysphagia, odynophagia, and fever. 8 cm mass in left lobe.	Euthyroid	Diabetes Mellitus	Histology proven plasma cell granuloma. Staining showed presence of IgG, IgM, and IgA.	Initial antibiotics. Surgical exploration with multiple biopsies
Martinez et al. 2002 [8]	46 F	Large painless neck mass. History of goitre.	Euthyroid	No	3 to 15 mm nodules separated by fibrous bundles. Numerous plasma cells with Hurtle cell changes	Total thyroidectomy
Mugler et al. 2003 [14]	46 M	Painless left neck mass. Family history of thyroid Ca. Dominant nodule on examination	Hypothroid	Hashimoto's	5 × 3 × 3 cm nodule. Changes consistent with thyroiditis, including Hurtle cell changes. Plasma cell aggregation, polyclonal on staining.	Neoplasm could not be ruled out on FNA. Total thyroidectomy
Talmi et al. 1989 [21]	51 F	Painless enlarging nodule in right lobe.	Not known	No	2 cm white nodule. Mature polyclonal plasma cells.	Lobectomy
Yapp et al. 1985 [9]	61 F	Painless goiter enlargement.	Hypothyroid	No	Polyclonal plasma cells. Hurtle cell changes. Some lymph node enlargement	Total thyroidectomy
Zingrillo et al. 1995 [10]	65 F	Neck swelling and breathing difficulties. 3 × 5 cm nodule in left lobe	Hypothyroid	Hashimoto's	Polyclonal plasma cells with lymphocytic infiltrate. Hurtle cell changes present	Total thyroidectomy
